# Hyperspectral Inversion of Soil Organic Carbon in Daylily Cultivation Areas of Yunzhou District

**DOI:** 10.3390/s26020740

**Published:** 2026-01-22

**Authors:** Zelong Yao, Xiuping Ran, Chenbo Yang, Ping Li, Rutian Bi

**Affiliations:** 1 College of Resources and Environment, Shanxi Agricultural University, Jinzhong 030801, China; 20232284@stu.sxau.edu.cn (Z.Y.);; 2 College of Agriculture, Shanxi Agricultural University, Jinzhong 030801, China

**Keywords:** daylily, soil organic carbon (SOC), soil texture, hyperspectral, machine learning

## Abstract

Accurate determination of Soil Organic Carbon (SOC), which is the foundation of soil health and safeguards ecological and food security, is crucial in local agricultural production. We aimed to investigate the influence of soil texture on hyperspectral models for predicting SOC content and to evaluate the role of different preprocessing methods and feature band selection algorithms in improving modeling efficiency. Laboratory-determined SOC content and hyperspectral reflectance data were obtained using soil samples from daylily cultivation areas in Yunzhou District, Datong City. Mathematical transformations, including Savitzky–Golay smoothing (SG), First Derivative (FD), Second Derivative (SD), Multiplicative Scatter Correction (MSC), and Standard Normal Variate (SNV), were applied to the spectral reflectance data. Feature bands extracted based on the successive projection algorithm (SPA) and Competitive Adaptive Reweighted Sampling (CARS) were used to establish SOC content inversion models employing four algorithms: partial least-squares regression (PLSR), Random Forest (RF), Backpropagation Neural Network (BP), and Convolutional Neural Network (CNN). The results indicate the following: (1) Preprocessing can effectively increase the correlation between the soil spectral reflectance process and SOC content. (2) SPA and CARS effectively screened the characteristic bands of SOC in daylily cultivated soil from the spectral curves. The SPA algorithm and CARS selected 4–11 and 9–122 bands, respectively, and both algorithms facilitated model construction. (3) Among all the constructed models, the FD-CARS-PLSR performed most prominently, with coefficients of determination (R^2^) for the training and validation sets reaching 0.93 and 0.83, respectively, demonstrating high model stability and reliability. (4) Incorporating soil texture as an auxiliary variable into the PLSR inversion model improved the inversion accuracy, with accuracy gains ranging between 0.01 and 0.05.

## 1. Introduction

Soil organic carbon (SOC) is a crucial component of arable ecosystems and serves as a core indicator of soil fertility and health. It directly influences crop growth and yield by regulating soil structure, microbial communities, nutrient cycling, and water retention capacity, thereby significantly affecting cultivated ecosystems and food security [1].

Yunzhou District, Datong City, is a typical daylily cultivation area, in which this industry has become a local specialty pillar [2]. However, in an ecologically fragile zone, the quality and yield of daylilies in Yunzhou District depend heavily on soil fertility, with SOC constituting a vital component. Therefore, acquiring SOC information enables the identification of degraded farmland, optimization of soil-water conservation measures, and enhancement of agroecosystem resilience. Accurate measurement of SOC in daylily cultivation areas provides scientific guidance for fertilization and soil improvement, thereby reducing cultivation costs, increasing value-added products, supporting farmer income growth, and sustainable industry development. Simultaneously, it facilitates the alignment of Yunzhou District with that of its “Dual Carbon” goals (carbon peak and carbon neutrality), promoting carbon trading, ecological compensation mechanisms, and green agricultural transformation.

The current SOC detection methods include potassium dichromate oxidation and dry combustion [3]. While offering high accuracy and reproducibility, these methods involve tedious procedures and require skilled personnel, thus failing to meet the precise agricultural demands [3]. Hyperspectral technology provides rapid and nondestructive monitoring. Numerous domestic and international studies used hyperspectral techniques for soil analysis. Song et al. [4] acquired three hyperspectral datasets (laboratory, in situ field, and UAV spectra) to model the SOC content in the Huangshui River Basin, Qinghai, and demonstrated SOC estimation capabilities across all data types. Yuan et al. [5] employed Partial Least Squares Regression (PLSR), SVR, and DNN algorithms to construct prediction models for total nitrogen (TN), total phosphorus (TP), and soil organic matter (SOM), achieving high inversion accuracy. Bai et al. [6] compared various band selection algorithms and developed an MSC-SVD-SVR model for high-precision TN inversion. These studies confirm the extensive application of hyperspectral technology in soil nutrient estimation.

However, most previous SOC prediction studies solely considered the mathematical transformations of full-spectrum data, followed by feature band selection using correlation coefficients or algorithms before modeling, focusing exclusively on SOC–spectral relationships [7]. As the SOC content is influenced by multiple factors (soil texture, pH, elevation, and soil type), incorporating auxiliary variables into inversion models is essential. For example: Zhao et al. [8] constructed a large-scale time-series reflectance composite dataset covering Bavaria, Germany, combining CNN deep learning with topographic features to significantly enhance regional SOC prediction accuracy. Ma et al. [9] achieved high-precision SOM modeling in Ebinur Lake Reserve by introducing EC, Fe, and pH as soil covariates. Tang et al. [10] incorporated elevation data to account for topographic influences on soil property estimation. Gu et al. [11] introduced terrain, climate, and remote sensing indices to develop soil particle-size estimation models using RF and XGBoost, with XGBoost demonstrating superior accuracy (R^2^ = 0.91). Jia et al. [12] utilized the RF, XGBoost, ERT, and LightGBM algorithms with DEM-derived variables for soil conductivity inversion in Pingluo County, Ningxia, where ERT achieved optimal performance (R^2^ = 0.96). These studies demonstrate that introducing auxiliary variables can enhance the precision of the inversion model.

Therefore, we targeted the SOC content in daylily cultivation soils in Yunzhou District, Datong City. Laboratory-acquired SOC content and hyperspectral reflectance data were subjected to multiple pre-processing algorithms. Feature bands were selected using Successive Projections Algorithm (SPA) and Competitive Adaptive Reweighted Sampling (CARS) to construct the SOC inversion models. Soil texture was subsequently incorporated to evaluate its impact on model performance with the aim of establishing hyperspectral estimation methods for daylily soil SOC. The research objectives were as follows:

(1) Investigation of the preprocessing effects on SOC spectral correlations.
(2) Identification of SOC-sensitive spectral bands.
(3) Development of rapid SOC estimation models.
(4) Evaluation of the influence of soil texture on estimation accuracy.

## 2. Materials and Methods

### 2.1. Study Area and Sample Collection

**Figure 1:**
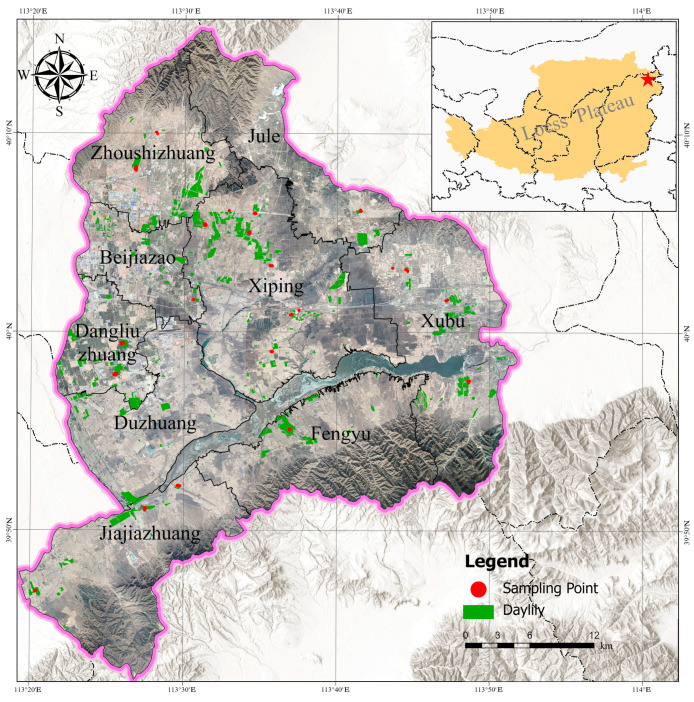
Study Area Delineation Map.

The study area (Figure 1) is located in Yunzhou District, Datong City, Shanxi Province (113°20′–113°55′ E, 39°43′–40°16′ N), with an average elevation of 1040 m. The region experiences mean annual temperature, precipitation, and frost-free period of 6.4 °C, 389 mm, and 125 days, respectively, and an annual accumulated active temperature of 2846.5 °C. Situated at the convergence of the Loess Plateau and the northern agro-pastoral ecotone, Yunzhou District comprises 730,000 mu (≈48,667 hectares) of cultivated land, with plain, hilly, and mountainous terrains accounting for 69%, 29%, and 2%, respectively. Primary crops include corn and potatoes, whereas daylilies (Hemerocallis citrina) are the dominant cash crop. In 2021, daylily cultivation reached 165,000 mu [2]. As a perennial herb, the daylily thrives under sunny, moist conditions and exhibits drought/poor soil tolerance. However, sufficient water and nutrients are essential throughout the growth cycle to ensure yield and quality.

During the distribution of sampling points, comprehensive consideration was given to factors such as daylily cultivation conditions, topography and landforms, and the distribution of townships. Sampling was conducted monthly from April to August 2024 across 60 sites. Surface soil samples (0–20 cm in depth) were collected using a soil auger following a five-point sampling method. After the manual removal of plant roots, the samples were homogenized, bagged, and sealed with site-specific labels. The GPS coordinates of the sampling centers were recorded. Samples were air-dried for 15 days in a ventilated indoor environment, ground, and sieved through a 0.15 mm nylon mesh.

### 2.2. Data Acquisition

The screened soil samples were evenly divided into two portions: one for the laboratory determination of organic carbon content and texture, and the other for hyperspectral data acquisition. Organic carbon content was measured using the potassium dichromate oxidation method. The soil texture composition was determined by laser diffraction analysis.

Hyperspectral data were collected using an ASD FieldSpec4 spectroradiometer (ASD Inc., Longmont, CO, USA, 350–2500 nm). The instrument features spectral sampling interval and resolution of 1.4 nm and 3 nm in the 350–1000 nm range, respectively, with corresponding values of 1.1 nm and 6 nm in the 100–2500 nm range. The soil samples were placed in black containers (3 cm radius and 4 cm depth) with leveled surfaces. Measurements were performed in a dark room using a 50 W halogen lamp (ASD Inc., Longmont, CO, USA) as the sole light source. The sensor probe was positioned 30 cm above the sample surface. A white reference calibration was performed prior to each measurement. Ten spectral readings were averaged for each sample to obtain the final reflectance spectrum.

### 2.3. Spectral Data Preprocessing

The bands within 400–2450 nm were selected as the original reflectance (R) for preprocessing. Five preprocessing methods were applied: Savitzky–Golay smoothing (SG), First derivative (FD), Second derivative (SD), Multiplicative scatter correction (MSC), and Standard normal variate (SNV) [13,14]. The SG employs local polynomial least-squares fitting to smooth the spectra and remove noise. The FD and SD were used to calculate the reflectance change rates between adjacent bands to eliminate the linear background interference from soil particle scattering or illumination variations. MSC corrects nonlinear scattering effects by separating chemical absorption signals from physical scattering noise. The SNV removes the multiplicative effects caused by particle scattering and path length differences, mitigating the effects of particle size distribution and surface roughness.

### 2.4. Feature Band Selection

#### 2.4.1. CARS

CARS implements Darwinian evolutionary principles through two mechanisms: an exponentially decreasing function and adaptive reweighted sampling. The initial band weights were assigned based on the PLS regression coefficients. The algorithm iteratively reduces the proportion of retained bands via exponential decay. Resample bands using weight-based probabilities. Monte Carlo cross-validated sub-models were evaluated using the root mean square error of cross-validation (RMSECV), with the minimal-RMSECV subset selected as the optimal features. This “survival of the fittest” approach eliminates redundant bands and enhances model robustness.

#### 2.4.2. SPA

SPA addresses multicollinearity through forward variable selection. Starting with the initial band, the process was performed iteratively. The remaining bands were projected onto selected band vectors. The band with the maximum projection vector length was added. This process continued until the preset band numbers were reached. Optimal bands were determined by selecting the subset yielding the minimal RMSE in multiple linear regression cross-validation.

### 2.5. Model Development

Four models were built for SOC inversion:

PLSR: Projects latent variables to eliminate spectral collinearity effective for high-dimensional data with limited samples. In the PLSR model of this study, the optimal number of latent variables (k) was determined through multiple iterations using 5-fold cross-validation, selecting the k-value that yielded the highest R^2^.

Random Forest (RF): Ensemble decision tree method for handling high-dimensional features and nonlinear relationships with strong anti-overfitting capabilities and feature importance assessment. In the RF model of this study, the number of decision trees was set to 100, and the minimum number of samples required at a leaf node was set to 2.

Backpropagation Neural Network (BP): A classic multilayer perceptron optimizing the weights via gradient descent for complex nonlinear mapping. In the BP neural network model of this study, the network architecture was configured as a single-hidden-layer feedforward network. The hidden layer contained 5 neurons. The maximum number of iterations was set to 1000, the target training error to 0.000001, and the learning rate to 0.01.

Convolutional Neural Network (CNN) extracts spatial features through convolutional layers and pooling operations, offering translation invariance and parameter-sharing advantages. The network architecture in this study employed three convolutional blocks for hierarchical feature extraction. The kernel sizes were 1 × 7, 1 × 5, and 1 × 3 for the successive blocks, with corresponding numbers of kernels being 16, 32, and 64. All convolutions used ‘same’ padding. Each convolutional layer was followed by Batch Normalization (BN) and a ReLU activation function. After the first two convolutional blocks, a 1 × 3 max-pooling layer (with a stride of 1 × 2) and a Dropout layer (rate = 0.3) were applied to mitigate overfitting. Subsequently, a global average pooling layer replaced large-scale fully connected layers. The final regression output for SOC was produced via a fully connected layer with one neuron. The model was trained using the Adam optimizer. The maximum number of epochs was set to 150, with a mini-batch size of 16 and an initial learning rate of 0.0005. A piecewise learning rate decay strategy was implemented, reducing the learning rate by a factor of 0.5 every 30 epochs. Furthermore, an L2 regularization coefficient of 0.001 and a gradient threshold of 1 were applied to enhance training stability.

### 2.6. Auxiliary Variables

In this study, soil texture was utilized as an auxiliary variable. The percentages of sand, silt, and clay content from the soil texture data were incorporated into the previously selected spectral bands to form a new composite matrix.

$$X=\left[X_{spectra},X_{texture}\right]$$

### 2.7. Model Evaluation and Implementation

Model performance was assessed using three metrics: the coefficient of determination (R^2^), root mean square error (RMSE), and residual prediction deviation (RPD).

## 3. Results and Analysis

### 3.1. Descriptive Statistics of SOC Content

**Figure 2:**
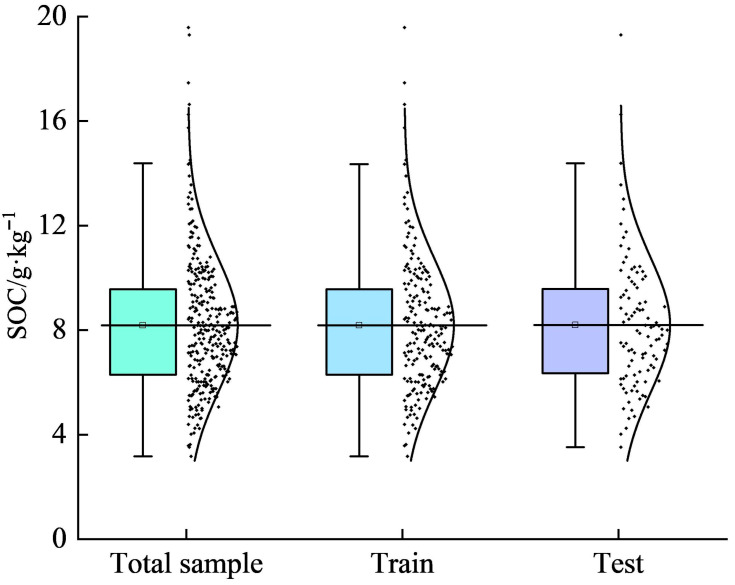
Descriptive statistics of SOC (g/kg). **Note:** Total sample refers to the complete set of samples; Train denotes the training set samples; and Test represents the test set samples.

To ensure a uniform SOC distribution between the training and validation sets, 300 soil samples were partitioned at a 2:1 ratio using the concentration gradient method, selecting 200 and 100 samples for training and validation, respectively. The data distributions of the total sample, training, and validation sets are shown in Figure 2. The total sample set had a minimum SOC of 3.17, maximum = 19.58, mean = 8.18, standard deviation of 2.56, and coefficient of variation (CV) of 0.31. The training and validation sets showed standard deviations of 2.55 and 2.58 with CV values of 0.31 and 0.32, respectively. All CVs exceeded 30%, indicating an approximately normal distribution suitable for hyperspectral SOC inversion modeling in daylily cultivation areas.

### 3.2. Analysis of Soil Spectral Reflectance Characteristics

**Figure 3:**
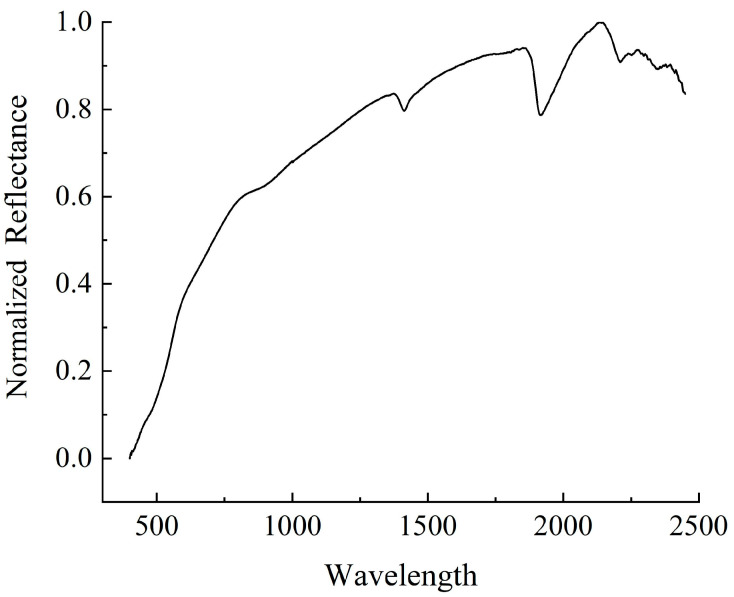
Mean normalized spectral curve of all soil samples.

In this study, a total of 300 soil hyperspectral reflectance curves were obtained. Figure 3 presents the mean spectral curve of all samples, which has been normalized. In the visible region (400–700 nm), the spectra display a gradual declining trend, forming a broad, shallow absorption trough at 450–570 nm, where the reflectance is significantly lower than that of the other visible segments. Within the near-infrared (NIR) range (700–1300 nm), the reflectance increased rapidly, with a relatively flat reflection peak at ~800–900 nm, characterized by a broad, blunt shape without sharp features. The short-wave infrared (SWIR) region (1300–2500 nm) constituted the core feature zone, exhibiting a steep absorption trough at 1400–1500 nm, the deepest full-band absorption trough at 1900–1950 nm, and a distinct double-peak structure between 2100 and 2200 nm. The SOC spectral response curves exhibited typical characteristics between 400 and 2500 nm.

### 3.3. Correlation Analysis and Feature Band Selection

**Figure 4:**
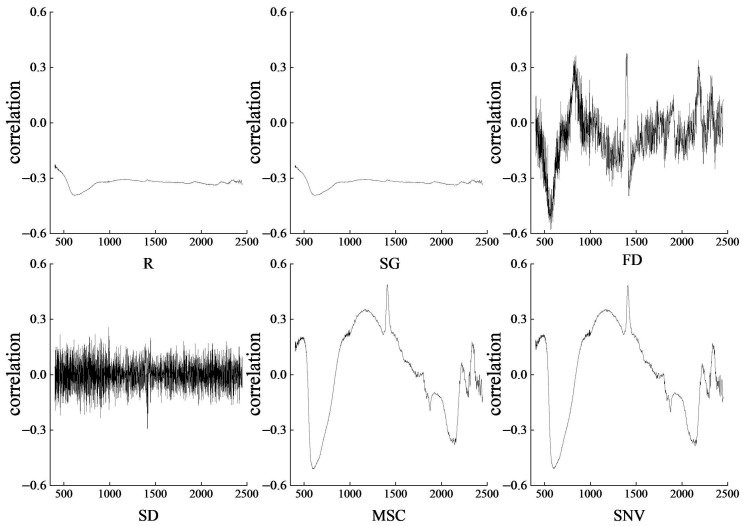
Correlation analysis of SOC with spectrally pretreated data.

The correlations between the SOC content and the raw and five preprocessed spectra are shown in Figure 4. The raw spectra exhibited predominantly negative correlation coefficients (maximum = −0.23, minimum = −0.39). SG smoothing maintained correlation trends similar to those of the raw spectra, without significant enhancement. FD processing improved correlations (max = 0.38, min = −0.581). Compared with that of FD, SD reduced fluctuations with coefficients ranging between −0.29 and 0.26, showing decreased SOC correlation. Both MSC and SNV scattering corrections performed comparably (max = 0.49/0.48, min = −0.51), outperforming raw data.

**Figure 5:**
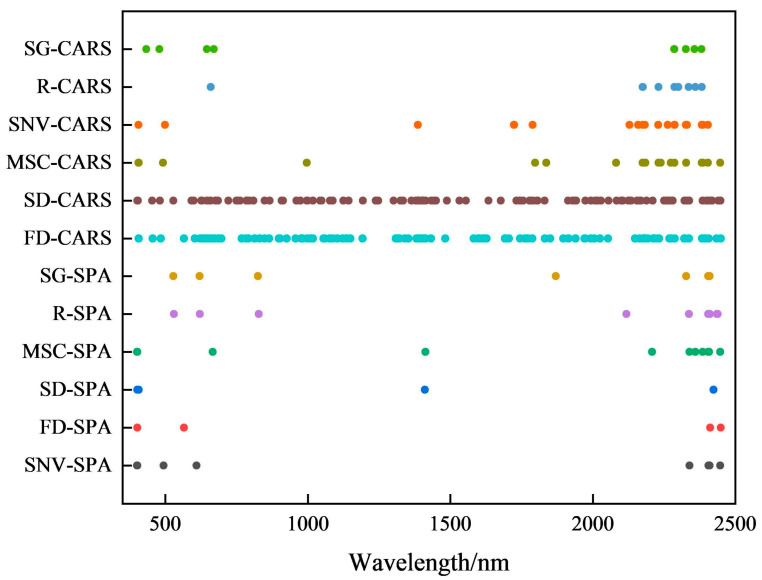
Characteristic wavelengths of SOC selected by SPA and CARS methods.

The CARS and SPA algorithms extracted the SOC feature bands from six preprocessed spectral datasets (Figure 5). SPA yielded relatively concise and discrete bands concentrated in the visible (400–800 nm) and SWIR (1400–2500 nm) regions, with the key bands (403, 2339, and 2405 nm) consistently selected across multiple preprocessing methods. CARS produced more continuous bands, particularly with FD/SD pre-processing, which yielded numerous bands distributed throughout the full spectrum. The other four preprocessing methods generated similar feature-band distributions, forming continuous clusters in the 2100–2500 nm SWIR range. Bands at 2175 nm, 2286 nm, and 2382 nm appeared frequently across the multiple preprocessing results.

### 3.4. SOC Content Model Construction

Based on full-spectrum bands (FULL), two feature band selection algorithms (SPA and CARS), and four modeling methods (PLSR, CNN, BP, and RF), we systematically constructed hyperspectral inversion models for soil SOC content in daylily cultivation areas of the Yunzhou District. The results are shown in Table 1. Algorithms such as full-spectrum bands, FD-CARS, and SD-CARS use excessive bands for modeling, leading to poor fitting performance and lower accuracy in the CNN model. A total of 72 models were established and analyzed. Comprehensively considering model accuracy and generalization ability, among the models built using full-spectrum bands, the MSC-PLSR and SNV-PLSR models performed best, with Rc^2^ values of 0.64 and 0.63, and Rv^2^ values of 0.68 and 0.67, respectively. Among the models constructed after SPA screening, MSC-BP demonstrated the best accuracy, with Rc^2^ and Rv^2^ values of 0.75 and 0.69, respectively. Most models built using the bands selected by CARS showed good accuracy and generalization ability, with the FD-PLSR model performing the best. The FD-CARS-PLSR model was also the best-performing model overall, achieving Rc^2^ and Rv^2^ values as high as 0.93 and 0.83, RMSE values of 0.69 and 1.07, and RPD values of 3.69 and 2.40, respectively. This excellent performance indicates that this model can effectively capture the complex relationship between soil SOC content and spectral data, while demonstrating extremely high model stability and reliability. The MSC-CARS-CNN model ranked next, achieving Rc^2^ and Rv^2^ of 0.90 and 0.71, respectively. However, models such as SNV-CARS-CNN and FD-CARS-RF exhibit significant overfitting problems. Although they achieved high Rc^2^ values of 0.93 and 0.91, their Rv^2^ values dropped sharply to 0.55 and 0.44, respectively. Particularly, the FD-CARS-RF model had an Rv^2^ below 0.5, with a decay rate as high as 51.65%. This severe overfitting suggests that substantial noise may have been introduced during model construction. Overall, among the 72 models established here, 22 achieved both Rc^2^ and Rv^2^ values greater than 0.6, demonstrating their strong capability to invert soil SOC content in the daylily cultivation areas of Yunzhou District.

To verify the agreement between the hyperspectral inversion results and the laboratory soil chemical analysis results, this study used the SOC content determined by the potassium dichromate oxidation method in the laboratory as the reference value (Measured SOC). The corresponding hyperspectral reflectance data of the same samples were preprocessed, subjected to feature band selection, and then input into the model to obtain the Predicted SOC. All samples were divided into a training set and a test set at a 2:1 ratio (200/100) using the concentration gradient method. The training set was used for model parameter calibration, while the test set served as independent data to test the model’s generalization capability and transferability.

**Figure 6:**
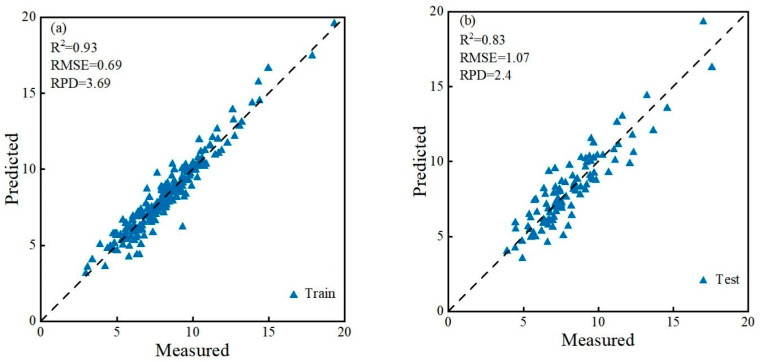
Comparison between predicted and measured SOC values under the FD-CARS-PLSR model. **Note:** Panel (**a**) shows the training set data, and panel (**b**) shows the test set data.

Taking the best-performing FD-CARS-PLSR model as an example (Figure 6), a one-to-one comparison was conducted between the predicted values and the chemically measured values. The scatter points were closely distributed along the 1:1 reference line, indicating a strong agreement between the model outputs and the laboratory measurements. Both the training set and the test set maintained high accuracy levels, demonstrating that the model not only fits well during the calibration stage but also retains high precision and stability when applied to an independent test set. The model effectively “replicates” the variation characteristics of chemically measured SOC, thereby enabling rapid and non-destructive estimation of SOC in the study area.

### 3.5. Impact of Soil Texture on SOC Content

The CNN, BP, and RF models exhibited unstable SOC inversion because of their low accuracy or poor generalization. Texture-based hyperspectral optimization models were developed using PLSR. Results (see table below) indicate that both underperforming (e.g., SD-SPA-PLSR) and high-performing (e.g., FD-CARS-PLSR) models improved in training set accuracy when incorporating texture (R^2^ increase: 0.01–0.05), confirming texture-SOC correlation. The validation accuracy fluctuated, which was attributed to the increased model complexity from the additional independent variables. From Table 2, it can be observed that, in the training set, the inclusion of soil texture led to varying degrees of increase in R^2^ and RPD for all models, alongside a decrease in RMSE. In the validation set, the R^2^, RMSE, and RPD values of different models showed mixed trends, with both increases and decreases. Apart from the SD + CARS series, which exhibited relatively significant fluctuations, the changes in R^2^ for other models all remained within 0.03.

## 4. Discussion

### 4.1. Impact of Pretreatment on Correlation

Existing studies proved that preprocessing hyperspectral curves can eliminate noise to some extent and enhance sensitivity to target indicators, thereby improving the correlation between spectral data and soil nutrients such as SOC. Ting et al. [15] performed 14 different transformations on the spectral curves of 174 soil samples collected from six different soil types. Their results showed that reciprocal, logarithmic, square root, and derivative transformations of spectral reflectance improved the relationship between reflectance and organic matter content to varying degrees, with the first derivative of the logarithm of reflectance exhibiting the highest correlation. Yanping et al. [16] used the derivative, continuous wavelet (CWT), and discrete wavelet (DWT) transformations to invert soil salt content (SSC). Their results indicated that FD combined with CWT and DWT effectively enhanced the correlation between SSC and hyperspectral data. Yang et al. [17] investigated the effect of a fractional-order derivative (FOD) on soil spectral reflectance by employing the UVE-PLSR algorithm combination to invert the soil total nitrogen content (STN). Their results showed that under the same number of fitting points, the correlation between STN content and FOD gradually increased as the derivative order increased from 0 to 1 but exhibited a trend of first decreasing and then increasing as the derivative order increased from 1 to 2. For the same derivative order, the correlation increased with the number of fitting points. We employed three categories, totaling five pretreatment methods, to process the acquired hyperspectral data. Considering the correlation results between the soil spectral curves and SOC, SG processing did not improve the correlation between the spectral curves and SOC; however, it removed noise from the spectral curves while preserving their original trends. Contrastingly, FD, SD, MSC, and SNV effectively enhanced the correlation between the soil spectral curves and SOC. Furthermore, Figure 4 shows that the correlation trends among the pretreatment methods for the same category are similar. For example, FD and SD are both baseline correction pretreatments. After processing, the correlation between the spectral curves and SOC fluctuated between positive and negative values, indicating that both can enhance the sensitivity to the SOC response. However, SD exhibited reduced fluctuation compared with that of FD, suggesting that while removing baseline drift, it may also weaken some information content, necessitating further consideration of its applicability based on specific modeling strategies. The trends of the two scatter-correction methods, MSC and SNV, were similar. Unlike baseline correction methods, the correlation of spectral curves processed with scatter correction also improved, although it did not fluctuate between positive and negative values. This indicates that they effectively eliminated the scattering effects in the spectrum caused by physical interference, such as particle size and moisture, thereby increasing the number and intensity of bands significantly correlated with SOC. Overall, the different pretreatment methods vary in their effectiveness in enhancing the correlation between the spectra and SOC. SG focuses on noise removal; FD and SD emphasize spectral feature enhancement; and MSC and SNV prioritize physical interference correction. Combining the statistical analysis results, it can be preliminarily determined that spectra pretreated with FD, MSC, and SNV comprise greater modeling potential.

### 4.2. Impact of Band Selection Algorithms on the Model

Soil hyperspectral data are voluminous, and the full spectrum contains not only a large amount of information irrelevant to the target indicator but also substantial redundant and collinear information [18]. These factors affect the modeling effectiveness. Common band selection algorithms include correlation coefficient method, modeling power method, principal component analysis loading method, successive projections algorithm (SPA), interval partial least squares (iPLS), uninformative variable elimination (UVE), genetic algorithm (GA), random forest (RF), and loading partial graph (LPG), among others. Studies proved that selecting feature bands during hyperspectral data modeling can reduce the computational load, simplify the model, and improve its inversion accuracy. For example, Hao et al. [18] modeled feature bands selected by the SPA and CARS methods and full bands simultaneously using a CNN model. The results showed that both algorithms improved the model accuracy to varying degrees. We used the SPA and CARS algorithms to select the SOC characteristic bands. SPA, a feature band extraction method proposed by Araújo et al., focuses on selecting bands contributing the most to the model through successive projections [19,20]. Contrastingly, CARS selects bands through competitive adaptive reweighted sampling. Its principle is based on Monte Carlo sampling to retain statistically significant band groups, emphasizing the spectral continuity and overall correlation. The selection process prioritizes global correlation with the target variable [21]. Owing to their different principles, bands extracted by SPA and CARS often possess strong local features and exhibit higher continuity, respectively, which aligns with those of our selection results. We used both the SPA and CARS algorithms to reduce the full spectral data to less than 6%, achieving effective dimensionality reduction of the spectral data. The selection results indicated that the vast majority of the selected bands were located at 400–850 nm and 2100–2400 nm. This finding is consistent with that reported by Bouslihim [22] and Lei et al. [23]. The reason for this may be that dark humus (humic acid, fulvic acid, etc.) has significant absorption in the blue (450 nm) and red (650 nm) light regions, leading to reduced reflectance. The 2100–2400 nm region is significant because SOC organic molecules contain various hydrogen-containing functional groups (-CH, -NH, and -OH), whose overtone and combination absorption bands are located near 2100–2400 nm. The modeling results in Table 1 show that compared with that of the full-spectrum modeling, models built using bands selected by the SPA algorithm showed significant improvement for MSC- and SNV-pretreated data, although the optimization effect was minimal for models with the other four pretreatments. Among the models, the full-spectrum model demonstrated the best accuracy, which is similar to that of the findings of Zhang et al. [24]. This may be because SPA selects a small number of feature bands for modeling, losing a significant portion of important spectral information compared with that of the full spectrum. However, in these models, the accuracy gap for most was not large, yet the number of modeling bands drastically decreased from 2049 to 9 or even 4, greatly improving the modeling efficiency. Here, models built using bands selected by the CARS algorithm showed better improvements than those built using the full spectrum. Some validation set accuracy improvements reached 0.39, for example, SD-CARS-BP and SNV-CARS-BP. CARS not only improved the modeling efficiency and accuracy, but also significantly enhanced the generalization ability of the model.

### 4.3. Comparison of Different Combined Modeling Algorithms

Selecting an appropriate regression model is crucial [25]. With the rapid development of computer science, traditional linear regression models can no longer meet the current demands, and many machine learning and deep learning models have emerged accordingly. Zhengfei et al. [26] utilized AM-normalized preprocessing combined with SPA, ultimately constructing an XGBoost model capable of accurately inverting SOC in the central Yunnan Plateau irrigation district. Yongshi et al. [27] used BPNN, SVM, RF, and PLSR to establish inversion models for SOC in limestone soils of karst areas, and the results indicated that all four models could be used for SOC inversion. We adopted multiple modeling methods: the traditional linear regression model Partial Least Squares Regression (PLSR), nonlinear machine learning models, Random Forest (RF) and Backpropagation Neural Network (BP), and nonlinear deep learning model Convolutional Neural Network (CNN). Based on the modeling results, the modeling outcomes for the FD- and SD-processed spectral data screened by the SPA and CARS algorithms differed significantly. Whether FD-SPA or SD-SPA, the validation set R^2^ for the four constructed models was less than 0.4, lacking practical significance. The reason for this may be that SPA is essentially a forward selection variable screening method selecting variables contributing the most to the model variance, wavelength by wavelength. Although derivative processing can eliminate baseline drift and separate overlapping peaks, it significantly amplifies high-frequency noise and reduces the signal-to-noise ratio. Within these noise-amplified data, SPA is highly prone to selecting discrete wavelength points containing substantial noise rather than continuous feature regions truly representing the chemical information. This results in models containing excessive noise and extremely poor generalization ability. Contrastingly, the models built using FD-CARS and SD-CARS achieved validation set R-^2^ values greater than 0.8. Among them, FD-CARS-PLSR achieved Rc^2^ and Rv^2^ as high as 0.93 and 0.83, respectively, making it the optimal inversion model. The bands selected by FD-CARS and SD-CARS may not be suitable for CNN modeling, possibly due to the excessive number of bands, leading to an overly complex model construction. All models built using RF achieved Rc^2^ values greater than 0.75; however, their Rv^2^ values were poor, exhibiting severe overfitting to varying degrees. Most models built using CNN and BP showed good accuracy on both the test and validation sets, and could be used for practical applications. Among the models built using PLSR, under the same preprocessing, all CARS-PLSR outperformed SPA-PLSR models, which is consistent with that of the findings of Wang et al. [28]. This is because PLSR relies on the global linear structure of the data and the bands extracted by CARS can provide PLSR with more stable and accurate global information, thus demonstrating better modeling performance in certain cases.

### 4.4. Applicability of CNN in SOC Prediction

Currently, many scholars have employed CNN models for soil property prediction using hyperspectral data. When applying CNN for the inversion and prediction of hyperspectral information, the core concept is to treat the continuous spectral reflectance curve as a one-dimensional sequential signal, learning local features of the spectral curve through convolutional kernels sliding along the wavelength dimension. This study found that when using CNN for modeling, the inversion accuracy was better with a smaller number of modeling bands. When the number of bands increased, overfitting occurred, and the modeling even failed when using the full spectrum. The reason may be attributed to the high autocorrelation and significant multicollinearity among different bands in soil hyperspectral data. With a larger number of spectral bands, certain noise may exist within the spectral data. The combined effect of these factors likely leads to instability in the CNN model when the number of bands is high.

### 4.5. Influence of Soil Texture on Hyperspectral Models

Soil texture is a key factor influencing SOC content and its hyperspectral modeling. Soil texture is primarily determined by the size composition of soil particles, including the proportions of sand, silt, and clay. The size and distribution of these particles directly affect the physical properties of the soil, such as porosity, aeration, and water retention capacity, which, in turn, influence the accumulation and decomposition processes of organic matter in the soil. This textural variation not only affects the actual SOC content, but also influences its manifestation in hyperspectral data. Research indicates that the effect of soil texture on SOC content varies across different soil types, posing challenges for hyperspectral modeling. Michael et al. indicated that the correlation between SOC content and hyperspectral reflectance is stronger in soils with a higher clay content and weaker in sandy soils [29]. This suggests that soil texture determines, to some extent, the relationship between SOC content and hyperspectral data, thereby affecting the accuracy and reliability of hyperspectral modeling.

The influence of soil texture on the hyperspectral data was mainly reflected in the spectral reflectance and absorption features. Differences in soil texture lead to variations in the surface roughness, pore structure, and water retention capacity. These changes in physical properties directly affect the acquisition and interpretation of hyperspectral data [30]. Soils with higher clay content typically have smoother surfaces and higher water retention capacity, resulting in lower reflectance and stronger absorption features in the hyperspectral data. Conversely, sandy soils have higher surface roughness and weaker water retention capacity and usually exhibit higher reflectance and weaker absorption features in their hyperspectral data. This difference necessitates consideration of the influence of soil texture in hyperspectral modeling to improve the model accuracy and generalization ability. Gholizadeh et al. [31] experimentally demonstrated that in hyperspectral data, changes in clay content caused a significant decrease in reflectance at specific wavelengths, whereas changes in sand content caused a significant increase in reflectance. This difference must be leveraged in hyperspectral modeling through feature selection and variable optimization to enhance the predictive capability of the model for SOC content. Furthermore, Castaldi et al. [32] noted that differences in soil texture also affect the noise level and spectral resolution of hyperspectral data, further increasing the complexity of hyperspectral modeling. In hyperspectral modeling, introducing auxiliary variables (such as soil texture) can significantly improve the prediction accuracy. Auxiliary variables provide additional information, helping models to better capture the complex relationship between SOC content and hyperspectral data. For example, Zhou et al. [33] found that considering the soil texture during modeling significantly improved the inversion results for soil salinity content in irrigation districts compared with that of the estimations based solely on all samples. This is because the soil texture provides crucial information regarding soil physical properties, which may not be directly obtainable from hyperspectral data. By introducing soil texture as an auxiliary variable, models can better explain variations in SOC content, thereby enhancing prediction accuracy. By comparing the results of the CARS and SPA band selection algorithms combined with the PLSR model, it was found that after introducing soil texture as an auxiliary variable, the R^2^, RMSE, and RPD metrics for both the training and validation sets improved. For instance, in the FD-CARS-PLSR model, Rc^2^ and Rv^2^ increased from 0.9260 to 0.9317 and 0.8252 to 0.8271, respectively, indicating that soil texture as an auxiliary variable can further enhance model performance.

## 5. Conclusions

This study focused on the soils of daylily cultivation areas in Yunzhou District, Datong City, Shanxi Province. By collecting soil samples and determining their SOC content and hyperspectral reflectance data, a systematic investigation was conducted on hyperspectral modeling optimization and SOC inversion based on soil texture. The main conclusions are as follows:

(1) Spectral preprocessing effectively enhanced the correlation between spectra and SOC. While Savitzky–Golay (SG) smoothing did not significantly improve correlations, it aided in noise reduction. First derivative (FD), multiplicative scatter correction (MSC), and standard normal variate (SNV) transformations all significantly improved the spectral response to SOC. The correlation coefficient increased to a maximum of 0.38 after FD processing, while the highest coefficients after MSC and SNV treatments reached 0.49 and 0.48, respectively.
(2) The successive projections algorithm (SPA) and competitive adaptive reweighted sampling (CARS) algorithm effectively screened SOC-sensitive feature bands. Band Acquisition Methods: SPA and CARS were applied to the original spectra and five preprocessed spectral sets for feature band extraction. Band Distribution Characteristics: SPA selected a smaller number of bands (4–11), which were discretely distributed in the visible (400–800 nm) and short-wave infrared (1400–2500 nm) regions. Bands such as 403 nm, 2339 nm, and 2405 nm repeatedly appeared across multiple preprocessing methods. CARS selected a larger number of bands (9–122), with a more continuous distribution, forming a distinct cluster in the short-wave infrared region (2100–2500 nm). Bands like 2175 nm, 2286 nm, and 2382 nm were high-frequency occurrences. Band Application: The selected feature bands were used to construct four machine learning models (PLSR, RF, BP, CNN), replacing full-spectrum modeling, thereby achieving data dimensionality reduction and improved modeling efficiency.
(3) By evaluating the three accuracy metrics (R^2^, RMSE, and RPD) and comparing the predicted versus measured values, the FD-CARS-PLSR model was identified as the optimal SOC inversion model. Without incorporating soil texture, this model achieved a training set R^2^ of 0.93, RMSE of 0.69, and RPD of 3.69. For the validation set, it attained an R^2^ of 0.83, RMSE of 1.07, and RPD of 2.40, demonstrating excellent predictive accuracy and generalization capability, making it suitable for rapid, non-destructive monitoring of SOC in daylily cultivation areas.
(4) Incorporating soil texture further improved the inversion accuracy of the PLSR model. After adding sand, silt, and clay content as auxiliary variables to the PLSR model, the training set R^2^ generally increased by 0.01–0.05, and validation set accuracy also improved. This indicates a correlation between soil texture and SOC, confirming that soil texture can serve as an effective covariate to enhance model robustness.

In summary, through hyperspectral preprocessing, feature band selection, and multi-model comparison, this study constructed a hyperspectral inversion model suitable for SOC prediction in the soils of Yunzhou’s daylily cultivation areas. It also verified the positive role of soil texture in improving model accuracy, providing technical support for regional cropland quality monitoring and carbon management.

## 6. Limitations and Future Research Directions

This study was conducted in the Yunzhou District of Datong City, with all sampling points located within its daylily cultivation areas. The model parameters may be jointly influenced by local soil types, farming practices, and climatic conditions. Consequently, its applicability to other regions or under different vegetation covers requires further testing through cross-regional sampling and independent external validation.

The sample collection for this study was confined to the period from April to August 2024. Although collections were conducted monthly, interannual variability in the soil properties of daylily fields was not considered. Future research could investigate the differences in SOC inversion using hyperspectral data from daylily cultivation soils across different years.

## Figures and Tables

**Table 1:** Accuracy Metrics of SOC Models Based on Hyperspectral Data.

		R_c_^2^	RMSE_c_	RPD_c_	R_v_^2^	RMSE_v_	RPD_v_			R_c_^2^	RMSE_c_	RPD_c_	R_v_^2^	RMSE_v_	RPD_v_
P L S R	R-SPA	0.59	1.57	1.56	0.63	1.68	1.65	B P	R-SPA	0.69	1.37	1.79	0.63	1.69	1.64
	SG-SPA	0.59	1.56	1.57	0.64	1.65	1.68		SG-SPA	0.72	1.29	1.90	0.64	1.67	1.66
	FD-SPA	0.37	1.95	1.26	0.36	2.20	1.26		FD-SPA	0.51	1.71	1.43	0.22	2.43	1.14
	SD-SPA	0.08	2.34	1.05	0.12	2.58	1.07		SD-SPA	0.14	2.27	1.08	0.03	2.71	1.02
	MSC-SPA	0.64	1.52	1.68	0.68	1.45	1.78		MSC-SPA	0.75	1.28	1.99	0.69	1.42	1.82
	SNV-SPA	0.58	1.64	1.56	0.70	1.41	1.84		SNV-SPA	0.72	1.36	1.88	0.68	1.45	1.78
	R-CARS	0.58	1.64	1.55	0.52	1.78	1.45		R-CARS	0.66	1.47	1.73	0.52	1.79	1.45
	SG-CARS	0.61	1.59	1.60	0.64	1.54	1.68		SG-CARS	0.73	1.32	1.93	0.68	1.45	1.78
	FD-CARS	0.93	0.69	3.69	0.83	1.07	2.40		FD-CARS	0.83	1.04	2.44	0.68	1.46	1.77
	SD-CARS	0.90	0.80	3.21	0.62	1.59	1.62		SD-CARS	0.80	1.13	2.26	0.54	1.75	1.47
	MSC-CARS	0.72	1.35	1.89	0.72	1.38	1.91		MSC-CARS	0.78	1.21	2.12	0.73	1.35	1.92
	SNV-CARS	0.67	1.45	1.76	0.61	1.60	1.61		SNV-CARS	0.76	1.24	2.05	0.61	1.59	1.62
	R-FULL	0.65	1.44	1.71	0.65	1.64	1.69		R-FULL	0.72	1.30	1.89	0.64	1.66	1.67
	SG-FULL	0.63	1.48	1.65	0.64	1.65	1.68		SG-FULL	0.66	1.43	1.71	0.62	1.70	1.63
	FD-FULL	0.71	1.31	1.87	0.51	1.93	1.43		FD-FULL	0.64	1.47	1.67	0.18	2.50	1.11
	SD-FULL	0.70	1.33	1.84	−0.03	2.80	0.99		SD-FULL	0.41	1.88	1.30	−0.33	3.18	0.87
	MSC-FULL	0.64	1.47	1.66	0.68	1.57	1.77		MSC-FULL	0.63	1.49	1.65	0.51	1.93	1.43
	SNV-FULL	0.63	1.48	1.65	0.67	1.59	1.75		SNV-FULL	−0.60	3.09	0.79	−0.17	2.98	0.93
C N N	R-SPA	0.83	1.00	2.40	0.45	2.09	1.36	R F	R-SPA	0.78	1.14	2.14	0.01	2.74	1.01
	SG-SPA	0.81	1.12	2.31	0.54	1.69	1.48		SG-SPA	0.78	1.14	2.14	0.05	2.69	1.03
	FD-SPA	0.72	1.30	1.90	0.16	2.48	1.10		FD-SPA	0.85	0.94	2.61	0.30	2.30	1.20
	SD-SPA	0.69	1.47	1.79	−0.46	2.88	0.83		SD-SPA	0.76	1.20	2.04	0.02	2.73	1.02
	MSC-SPA	0.90	0.83	3.14	0.67	1.40	1.75		MSC-SPA	0.85	0.99	2.62	0.56	1.63	1.51
	SNV-SPA	0.89	0.85	2.97	0.57	1.71	1.53		SNV-SPA	0.87	0.92	2.77	0.52	1.79	1.44
	R-CARS	0.74	1.30	1.97	0.37	2.01	1.27		R-CARS	0.77	1.21	2.11	0.05	2.50	1.03
	SG-CARS	0.78	1.24	2.13	0.44	1.78	1.34		SG-CARS	0.79	1.17	2.18	0.06	2.48	1.04
	FD-CARS	0.11	0.91	1.06	0.06	1.01	1.03		FD-CARS	0.91	0.78	3.28	0.44	1.92	1.34
	SD-CARS	0.16	0.88	1.09	−0.03	1.05	0.99		SD-CARS	0.87	0.93	2.73	0.15	2.36	1.09
	MSC-CARS	0.90	0.74	3.20	0.71	1.55	1.86		MSC-CARS	0.89	0.84	3.02	0.39	2.00	1.29
	SNV-CARS	0.93	0.70	3.78	0.55	1.60	1.49		SNV-CARS	0.89	0.86	2.97	0.40	1.99	1.30
	R-FULL	−0.76	1.29	0.75	−0.68	1.27	0.77		R-FULL	0.83	1.02	2.41	0.05	2.68	1.03
	SG-FULL	−1.59	1.56	0.62	−1.47	1.54	0.64		SG-FULL	0.83	1.00	2.44	0.08	2.65	1.05
	FD-FULL	−0.02	0.98	0.99	−0.05	1.00	0.98		FD-FULL	0.91	0.73	3.35	0.35	2.23	1.24
	SD-FULL	−0.57	1.22	0.80	−0.71	1.28	0.77		SD-FULL	0.86	0.93	2.64	0.04	2.70	1.03
	MSC-FULL	−2.08	1.71	0.57	−1.95	1.68	0.58		MSC-FULL	0.90	0.77	3.19	0.40	2.13	1.30
	SNV-FULL	−1.81	1.63	0.60	−1.68	1.60	0.61		SNV-FULL	0.72	1.30	1.88	0.31	2.29	1.21

**Note:** Calibration (C) and Validation (V) represent the training set and test set, respectively. R^2^ denotes the coefficient of determination of the model, RMSE stands for the root mean square error, and RPD refers to the ratio of performance to deviation (hereafter).

**Table 2:** Accuracy metrics results of optimized models based on the PLSR model.

		R_c_^2^	RMSE_c_	RPD_c_	R_v_^2^	RMSE_v_	RPD_v_	∆R_c_^2^	∆RMSE_c_	∆RPD_c_	∆R_v_^2^	∆RMSE_v_	∆RPD_v_
C A R S	R	0.58	1.64	1.55	0.52	1.78	1.45	0.05	−0.10	0.10	0.01	−0.02	0.01
	R + Texture	0.63	1.54	1.65	0.53	1.76	1.47						
	FD	0.93	0.69	3.69	0.83	1.07	2.40	0.01	−0.03	0.15	0.00	−0.01	0.01
	FD + Texture	0.93	0.66	3.84	0.83	1.07	2.42						
	SD	0.90	0.80	3.21	0.62	1.59	1.62	0.02	−0.11	0.50	−0.10	0.20	−0.18
	SD + Texture	0.93	0.69	3.71	0.51	1.79	1.44						
	SG	0.61	1.59	1.60	0.64	1.54	1.68	0.01	−0.03	0.03	−0.03	0.07	−0.07
	SG + Texture	0.62	1.57	1.63	0.61	1.61	1.61						
	MSC	0.72	1.35	1.89	0.72	1.35	1.91	0.02	−0.04	0.06	0.00	0.01	−0.01
	MSC + Texture	0.73	1.31	1.95	0.72	1.36	1.90						
	SNV	0.67	1.45	1.76	0.61	1.60	1.61	0.03	−0.06	0.08	−0.03	0.05	−0.05
	SNV + Texture	0.70	1.39	1.84	0.59	1.65	1.56						
S P A	R	0.59	1.57	1.56	0.63	1.68	1.65	0.02	−0.03	0.03	0.02	−0.06	0.06
	R + Texture	0.60	1.54	1.59	0.65	1.63	1.71						
	FD	0.37	1.95	1.26	0.36	2.20	1.26	0.01	−0.01	0.01	−0.01	0.02	−0.01
	FD + Texture	0.37	1.94	1.27	0.35	2.23	1.25						
	SD	0.08	2.34	1.05	0.12	2.58	1.07	0.01	−0.01	0.01	−0.02	0.02	−0.01
	SD + Texture	0.09	2.33	1.05	0.11	2.60	1.06						
	SG	0.59	1.56	1.57	0.64	1.65	1.68	0.01	−0.02	0.03	0.01	−0.03	0.03
	SG + Texture	0.61	1.53	1.60	0.65	1.62	1.71						
	MSC	0.64	1.52	1.68	0.68	1.45	1.78	0.02	−0.04	0.04	−0.01	0.03	−0.03
	MSC + Texture	0.66	1.48	1.72	0.67	1.48	1.74						
	SNV	0.58	1.64	1.56	0.70	1.41	1.84	0.01	−0.03	0.03	−0.02	0.04	−0.05
	SNV + Texture	0.60	1.61	1.58	0.68	1.45	1.78						

## Data Availability

The data presented in this study are available on request from the corresponding author (since this data originates from an academic research project, it must not be used for any non-academic research purposes.)
